# Optimizing qPlus sensor assemblies for simultaneous scanning tunneling and noncontact atomic force microscopy operation based on finite element method analysis

**DOI:** 10.3762/bjnano.8.70

**Published:** 2017-03-20

**Authors:** Omur E Dagdeviren, Udo D Schwarz

**Affiliations:** 1 Department of Mechanical Engineering and Materials Science, Yale University, New Haven, CT 06520, USA; 2 Center for Research on Interface Structures and Phenomena (CRISP), Yale University, New Haven, CT 06520, USA; 3 Department of Chemical and Environmental Engineering, Yale University, New Haven, CT 06520, USA

**Keywords:** force sensor, noncontact atomic force microscopy, quartz tuning forks, scanning tunneling microscopy, self-sensing probe

## Abstract

Quartz tuning forks that have a probe tip attached to the end of one of its prongs while the other prong is arrested to a holder (“qPlus” configuration) have gained considerable popularity in recent years for high-resolution atomic force microscopy imaging. The small size of the tuning forks and the complexity of the sensor architecture, however, often impede predictions on how variations in the execution of the individual assembly steps affect the performance of the completed sensor. Extending an earlier study that provided numerical analysis of qPlus-style setups without tips, this work quantifies the influence of tip attachment on the operational characteristics of the sensor. The results using finite element modeling show in particular that for setups that include a metallic tip that is connected via a separate wire to enable the simultaneous collection of local forces and tunneling currents, the exact realization of this wire connection has a major effect on sensor properties such as spring constant, quality factor, resonance frequency, and its deviation from an ideal vertical oscillation.

## Introduction

Scanning tunneling microscopy (STM) [1] and non-contact atomic force microscopy (NC-AFM) [1–3] are powerful methods allowing the visualization of the atomic structure of a surface, with STM probing the electronic properties of the sample and NC-AFM its chemical nature with picoampere, piconewton, and picometer resolution [4–11]. Thereby, STM relies on measuring a tunneling current collected by a conducting tip located in close proximity of the probed surface while NC-AFM uses the perturbation that surface forces impose on the vibration of a cantilever to sense the proximity of the surface from a tip located at the end of the cantilever [12–14]. It is even possible to conduct simultaneous STM and NC-AFM experiments, which deliver complementary information, when a conducting probe is attached to the end of the oscillator [5–11].

Towards this end, microfabricated cantilevers [15–17], length-extension resonators [18–20], and quartz tuning forks in the so-called “qPlus” configuration, in which one of the prongs of the fork is allowed to vibrate freely while the other one is attached onto a holder [19,21], have previously been used to conduct combined STM/AFM experiments. Among these, quartz tuning forks in qPlus configuration have gained the widest popularity as they offer several advantages such as self-sensing properties, low cost, a freedom in the selection of the materials used as local probes, and physical dimensions that allow experimentalists to assemble sensors right in their own labs with relative ease [19,22–25]. But even though this in-lab assembly of qPlus sensors is a manageable task, the small size of the tuning forks (about 3 mm) and complexity of the overall sensor architecture, in particular when outfitted with a separate wire connection to the probe tip for combined STM/AFM experiments, impedes the assembly of such sensors in a reliable and repeatable way for personnel without considerable experience. As a result, personal skills have often a major impact on the sensing characteristics of the completed device.

To help minimizing the related problems, this work investigates the influence of different tip mounting options on the spring constant, Q-factor, resonance frequency, and perturbation of the ideal vertical oscillation behavior using the finite element method (FEM). Building on an earlier study that quantified the performance of qPlus sensors without tips as a function of the location and amount of epoxy glue used to mount the fork onto its holder [26], we model in this work sensor assemblies that include tips. This approach allows one to conveniently reveal the evolution of the sensor performance as a function of the various choices that have to be made during assembly such as glue thickness and choosing the location where to attach the tip in the first place. For example, we find that spring constant, Q-factor, and eigenfrequency are attenuated for tip-holder setups that feature an increasing degree of asymmetry. This effect is, however, modest if compared to the effect of an asymmetric wire connection, as they are frequently added to collect a tunneling current for combined STM/NC-AFM measurements. Our calculations show that a poorly implemented connection could significantly increase the spring constant of the sensor, which leads to an underestimation of tip–sample interactions forces in local spectroscopy, and induce unwanted lateral motion that may affect the lateral resolution of the setup. As a consequence, establishing a highly symmetric tunneling connection with the smallest possible stiffness should receive highest priority during the assembly of tuning fork-based sensors.

## Methods

The results presented in the paper expand on a previously introduced approach for the FEM modeling of a tip-less, but otherwise complete qPlus-style sensor setup [26]. This previous model included a tuning fork of length *L* = 2426.3 µm, width *w* = 130.7 µm, and thickness τ = 234.1 µm mounted with epoxy glue on a holder made of Macor, with material choices and sensor geometry closely reflecting the design that we actually use in our lab. While in [26] the thickness of the epoxy layer and its overlap with the tuning fork (referred therein as parameters ‘*e*_thick_’ and ‘*e*_over_’, respectively) were varied to uncover their influence on the sensor properties, we keep them constant throughout this work at typical values of *e*_thick_ = 100 µm and *e*_over_ = 40 µm to solely focus on the effect the tip attachment has.

All calculations were carried out using the COMSOL Multiphysics 4.4 structural mechanics software package (COMSOL Multiphysics GmbH, Berlin, and Germany). Table 1 summarizes the values used for Young’s modulus *E*, the density ρ, Poisson’s ratio υ, and the damping coefficient η for all materials considered in the modeling, with values for quartz, epoxy glue, and Macor chosen as in [26] while the ones for gold and tungsten were taken from the material library of the simulation software [27]. Also note that (i) due to the comparatively low internal damping occurring inside Macor, gold, and tungsten, we do not assign a damping coefficient to any of these materials to speed up the calculations, and that (ii) the sensor is oscillating in vacuum; for experimentation in air, we would have to expect considerable additional viscous damping [28].

**Table 1:** Material properties used for finite element calculations. Since significant damping occurs only inside the quartz and, in particular, the epoxy glue, no damping coefficient is defined for Macor, gold, and tungsten.

material constant	quartz	epoxy glue	Macor	gold	tungsten

Young`s modulus (GPa)	82	7	300	79	411
Poisson’s ratio	0.17	0.35	0.22	0.42	0.28
mass density (kg/m^3^)	2648	1600	3900	19300	19250
damping coefficient	2 × 10^−4^	5 × 10^−3^	**—**	**—**	**—**

**Figure 1:**
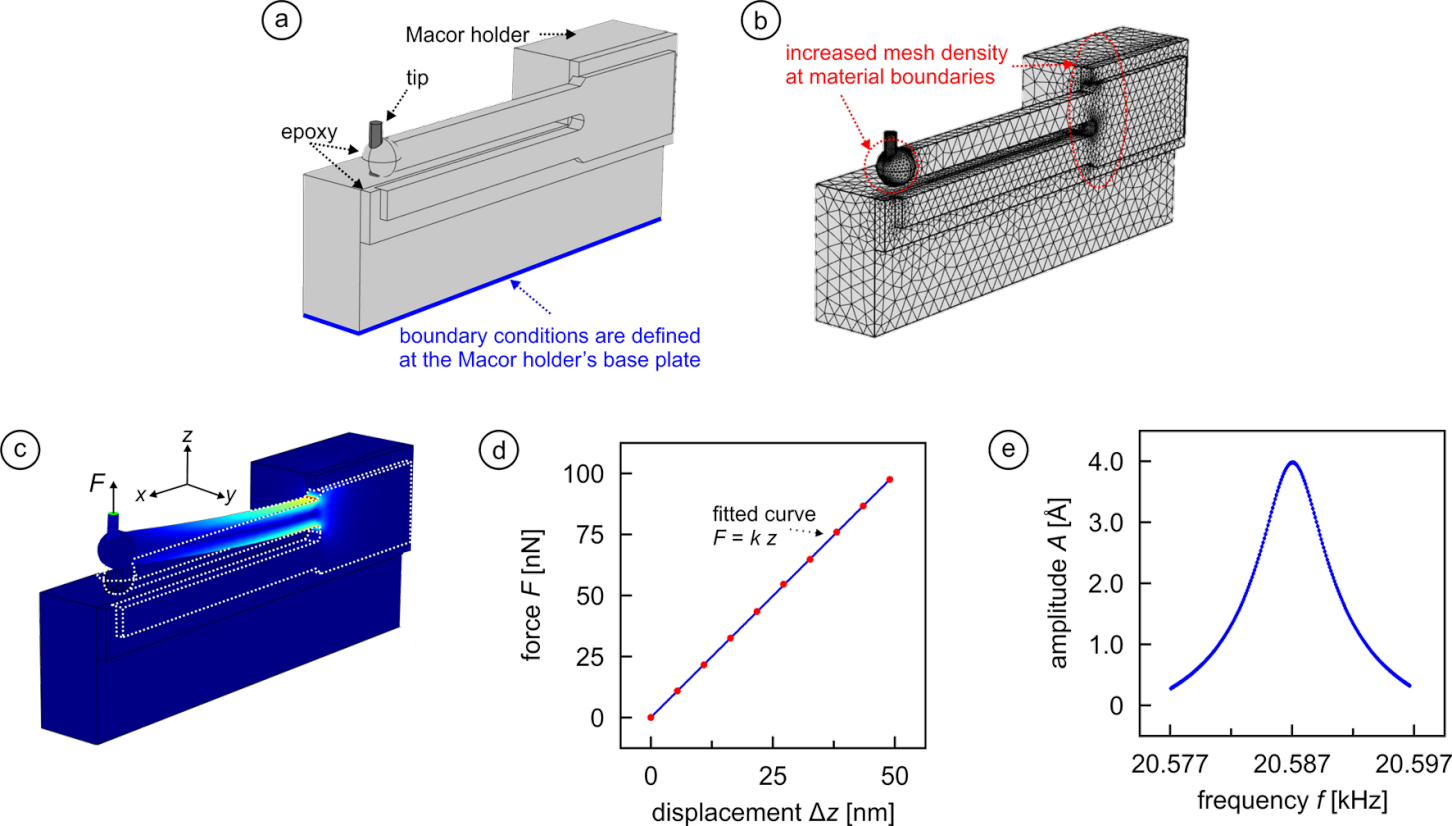
Figure explaining the model and procedures used throughout the paper. (a) Perspective sketch of all parts that were included into the model: tuning fork, Macor holder, epoxy glue and tip. (b) Map of the mesh distribution implemented in the finite element software during the calculations. (c) False color representation of local stress levels for the case where the free prong deforms under the influence of a static force *F* that is applied to the tip end (see arrow), with brighter colors representing higher stress. (d) Plot of force vs displacement data set from which the spring constant *k* is obtained by curve fitting. (e) Data set obtained from dynamical simulations (i.e., by applying an oscillatory displacement of varying frequency at the holder base plane highlighted in panel a) from which *f*_0_ and *Q* can be determined.

The model setup used for the FEM calculations is illustrated in Figure 1a. As in the earlier model of [26], boundary conditions for determining spring constant, quality factor, resonance frequency, and perturbation of the ideal vertical oscillation behavior are applied at the base of the Macor holder. The difference from this arrangement to the previous one is that we added a tungsten tip that is attached to the free prong using a spherical drop of epoxy glue. To save on computational costs, we represent the tip by a blunt rod of 100 µm diameter and 400 µm length, i.e., without shaping its end into an actual apex. In the panel, the location of the tip is depicted at the end of the free prong with the tip oriented straight up and being held in place by a spherical drop of glue with 150 µm radius. But in the course of the calculations, the location and orientation of the tip will be modified along with the amount of epoxy used for attaching the tip.

In panels (b–e) of Figure 1, we then establish some benchmark values for the qPlus sensor with tips. Figure 1b visualizes the mesh distributions used for the calculations. As before, mesh densities are increased at material boundaries and locations where particularly high stress is expected [29–31]. Figure 1c exposes areas of high stress when a constant force *F* is applied at the tip apex, with bright colors reflecting higher stress. From a measurement of the resulting *z* displacement for a series of different *F* values (Figure 1d), we can determine the spring constant *k* of the free prong to 1920 N/m. This is a little softer than the previously found value for a sensor without tip of 2021 N/m for two reasons: 1) The prong is now longer, which gives rise to higher *z* displacements; and 2) since the force pulls on the tip apex rather than then prong, some deformation will occur in the tip and, in particular, inside the glue. To determine quality factor *Q*, eigenfrequency *f*_0_, and perturbation Δ*y*/Δ*z* of the first eigenmode oscillation from motion in the main *x*–*z* oscillation plane, an oscillatory displacement of varying frequency along the *z*-direction is applied to the otherwise rigid holder base plane (cf. Figure 1a) while the motion of the end of the free prong is tracked as a function of *x*, *y*, and *z* (see [26] for details). Analyzing the respective data set presented in Figure 1e, this yields *Q* = 3874 and *f*_0_ = 20,592 Hz. Compared to the previously found values of *Q* = 3707 and *f*_0_ = 32,149 Hz without tip, we see that while the quality factor slightly increases, the main effect is that the additional mass of the tip and the glue causes a reduction of the resonance frequency by about 36%.

## Results and Discussion

### Effect of attaching a tip to the free prong of the tuning fork

In this section, we are investigating how different choices to attach the tip to the free prong of the qPlus sensor affect the evolution of the spring constant *k*, the first eigenfrequency *f*_0_, the quality factor *Q*, and degree by which the oscillation deviates from movement in the vertical *x*–*z* axis (the “perturbation” Δ*y*/Δ*z*). To assess the significance of these changes on the sensing capabilities of the device, let us recall from the discussion in [26] that high-resolution measurements involving qPlus sensors are to date mostly conducted in frequency modulation (FM) mode, where the reduction of the eigenfrequency *f*_0_ upon approach to the surface is the measured quantity (the so-called “frequency shift” Δ*f*) [32]. Since Δ*f* 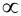 *f*_0_/*k* [33–36], we have to weight variations in *f*_0_ and *k* combined rather than individually. This is in particular important as any change in *f*_0_ is an indication that some change in *k* may have taken place as well, as *f*_0_ and *k* are entangled properties [26]. In contrast, the thermal noise δ*f*_thermal_ of the measurement, which is one of the main noise sources in FM-AFM, scales with *Q*^−1/2^, which is why an increase in quality factor is always desirable [32,37]. Finally, since a high ratio of Δ*y*/Δ*z* may introduce a non-negligible uncertainty in the accuracy of local measurements, this ratio is ideally as small as possible. Note, however, that for other ways of driving the measurements (such as in the recently introduced tuned-oscillator mode [38]), other priorities for the evolution of *f*_0_, *k*, and *Q* may exist.

**Figure 2:**
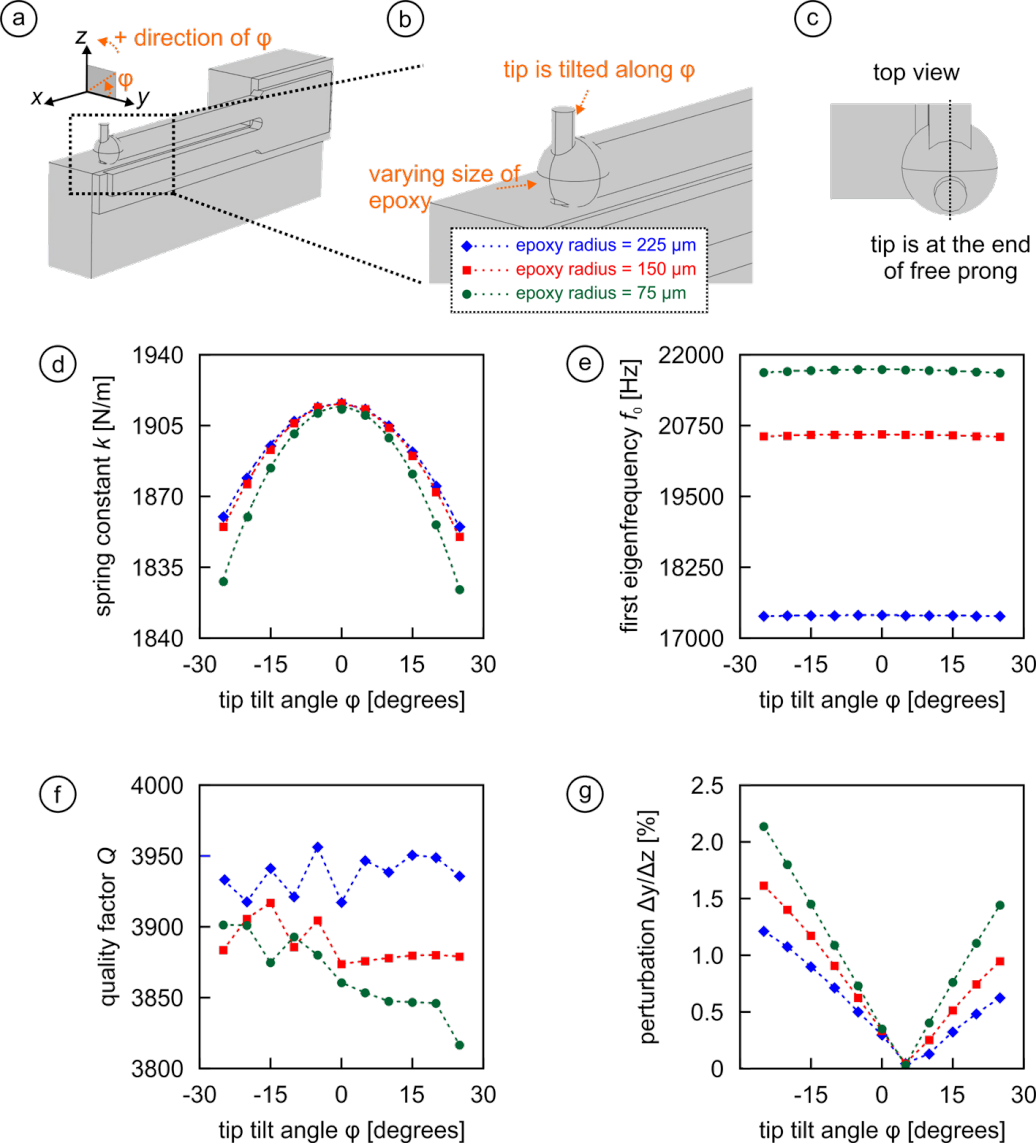
Effect of the tip tilt angle φ on *k*, *f*_0_, *Q*, and Δ*y*/Δ*z* for three different epoxy amounts used to attach the tip to the end of the free prong of the fork (green: 75 µm; red: 150 µm; blue: 225 µm). (a–c) Illustration of the geometrical arrangement and definition of the angle φ. (d) Increasing tip tilt results in decreasing spring constants regardless of the epoxy amount used. (e) The additional mass that goes along with larger amounts of glue reduces the eigenfrequency of the sensor significantly, but almost no effect of the tip tilt is detected. (f) *Q* changes by less than 0.1% within the screened parameter space. (g) Due to the assembly asymmetry introduced by tip tilting, the perturbation grows to over 2% for large tilt angles.

The first attachment choice studied in this paper is depicted in Figure 2a–c. Here, the blunt tungsten rod of 100 µm diameter and 400 µm length standing in for the tip is mounted at the end of the free prong aligned with the vertical symmetry axis of the prong and held by spherical blobs of epoxy glue with three different radii: 75 µm (green color in Figure 2d–g), 150 µm (red), and 225 µm (blue). For each of these three cases, the angle φ that the tip deviates from a fully vertical orientation (referred to as the ‘tip-tilt angle’) is varied in steps of 5° from −25° to +25° (see Figure 2a,b for illustration).

The effect of the different combinations is presented in Figure 2d–g. From Figure 2d we see that the spring constant *k* decreases up to 5% for tilted tips, which is consistent with results from an earlier numerical analysis [31]. Interestingly, the amount of glue does not have any effect on the spring constant as long as the tip is vertically oriented. For tilted tips the spring softens more for lesser amounts of epoxy since more epoxy stabilizes the tip more (remember that the force is applied at the tip end). In contrast, the first eigenfrequency of the sensor drops significantly with the amount of glue used due to the added mass, but it is almost inert to probe asymmetry (Figure 2e). Moreover, the quality factor changes by less than 0.1% with either epoxy amount or angle φ (Figure 2f). Finally, the perturbation Δ*y*/Δ*z* may increase to over 2% for the largest tilt angles φ (Figure 2g), with the smallest amount of glue showing the highest perturbations as the center of mass moves back towards the central symmetry axis of the prong the more glue is being used. We note in particular that due to the fact that the tuning fork is glued onto the holder from the side, the perturbation has its minimum at φ ≈ +5°. This is because at this angle, the asymmetry introduced by the tip approximately offsets the intrinsic perturbation induced by the non-symmetric attachment (cf. Figure 2e in [26]).

**Figure 3:**
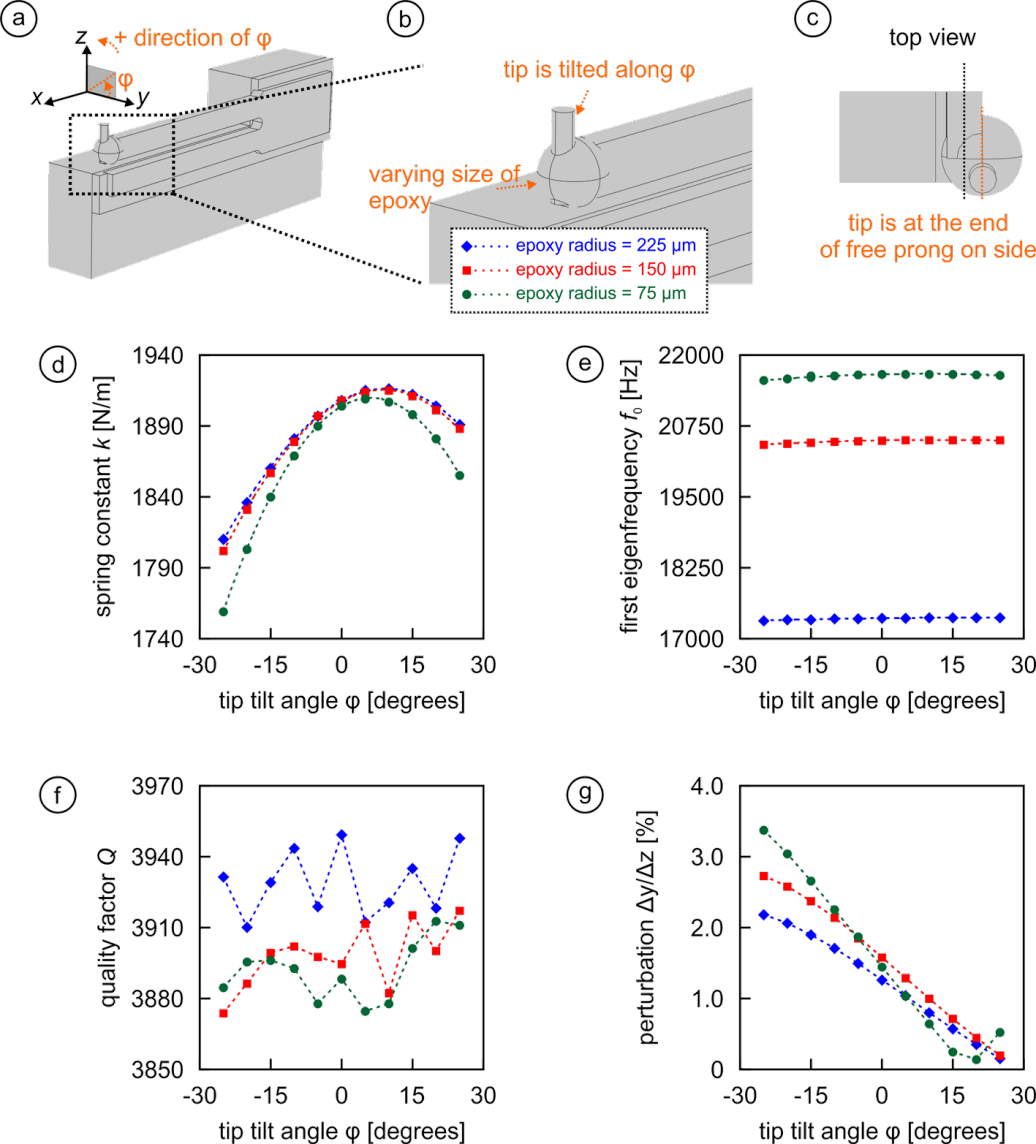
(a–c) Geometrical arrangement and definition of the angle φ for a configuration where the tip is not mounted axially symmetric to the central *x*–*z* plane of the free prong, but rather aligned with the far side of the prong when looked at from the holder. (d–g) Effect of the tip tilt angle φ on *k*, *f*_0_, *Q*, and Δ*y*/Δ*z* for three different epoxy amounts.

For the second configuration of attachment (Figure 3a–c), we essentially use the same setup as in case one (i.e., tracking the tip tilt angle φ for spherical epoxy glue blobs of 75 µm, 150 µm, and 225 µm sphere radius), but the twist is that the tip (and thus the sphere) is not aligned axially symmetric with the central *x*–*z* plane of the free prong any more as it was in case one (Figure 2c). Instead, it is now aligned with the side face of the prong that is away from the holder, as illustrated in Figure 3c. The main effect of attaching the tip “on the side” of the end of the prong is that it introduces additional asymmetry, the effects of which are presented in Figure 3d–g. First, the spring constant *k* (Figure 3d) follows the same trends as in Figure 2d, but the high point for *k* is now reached at φ ≈ 5–7° rather than φ = 0° to compensate for the added asymmetry. The results for the first eigenfrequency of the sensor assembly (Figure 3e) reproduce pretty much the dependency and values found in Figure 2e, as *f*_0_ is mainly dependent on the total mass added to the end of the prong rather than on its location or geometrical shape. Similarly, we find again that the quality factor is not modified by either epoxy amount or angle φ (Figure 3f). The mass unbalance, however, visibly affects the perturbation results (Figure 3g), where minimal deviations are found for φ ≈ 20–25° and values above 3% are reached. As a result, for typical NC-AFM peak-to-peak oscillation amplitudes of around 2 Å [38–39] lateral deviations larger than 6 pm may occur, which may be detectable but appears still negligibly small for most practical purposes.

**Figure 4:**
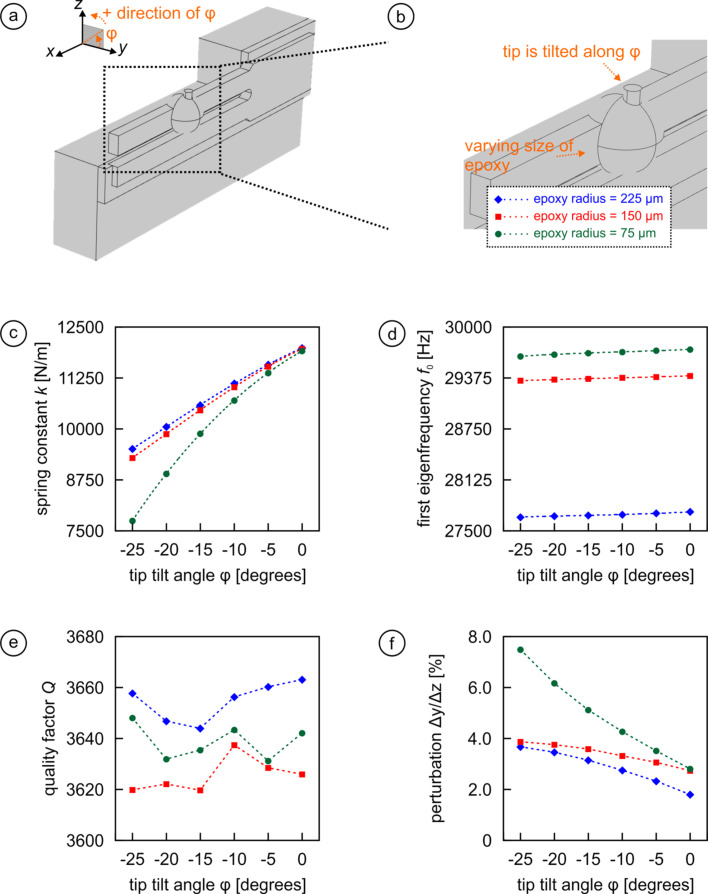
Model for a configuration where the tip is mounted at the half-length point of the free prong at the far side of the prong when viewed from the side of the holder. (a,b) Three-dimensional representations illustrating the geometrical layout. (c–f) Effect of tip tilt on *k*, *f*_0_, *Q*, and Δ*y*/Δ*z* for three different epoxy amounts.

To get a complete picture of the possibilities, we investigated in the third configuration a situation where the tip is not attached at the end of the prong, but rather at half-length of the prong at the far side of the prong if viewed from the holder (see Figure 4a,b). For consistency, the tip is again mounted by spherical blobs of glue featuring the same radii as above. The first thing we note is that the spring constant increases from ca. 2,000 N/m to ca. 12,000 N/m (Figure 4c). Asymmetry has also a far greater effect on the spring constant, with reductions of more than 30% for the case with the least glue used (note that the tip can only tilt towards negative values of φ as the prong blocks tilting into the positive direction). Since the additional mass provided by the tip and the glue is now closer to the clamped end of the prong, the resonance frequency drops less than before compared to the value without tip of *f*_0_ = 32,149 Hz, namely to values between 27,500 and 30,000 Hz depending on how much glue has been used (Figure 4d). But as before, *f*_0_ changes only minimal with varying tip tilt. The quality factor of the sensor decreases only insignificantly (around 5%; cf. Figure 4e) compared to the previous two configurations. Because the overall length of the prong is still the same; we would expect to see a dramatic change only if we were actually cutting the length of the prong in half as well. As before, *Q* is also only minimally affected by tip tilt. On the other hand, due to the substantial asymmetry induced by mounting the tip “on the side”, the perturbation from an ideal vertical modulation increases considerably to more than 2% even for straight up tip orientations (φ = 0°) and may reach values of 8% for small epoxy amounts and large tip tilts.

### Effect of a wire for collecting the tunneling current

In many qPlus sensor designs, a separate wire connects to the metallic probe tip to allow for an independent collection of a tunneling current between tip and sample for simultaneous STM/NC-AFM measurements [11,38,40]. It is intuitively clear that such a wire must have an appreciable influence on the properties of the sensor. This section therefore attempts to quantify this effect for two basic model geometries.

**Figure 5:**
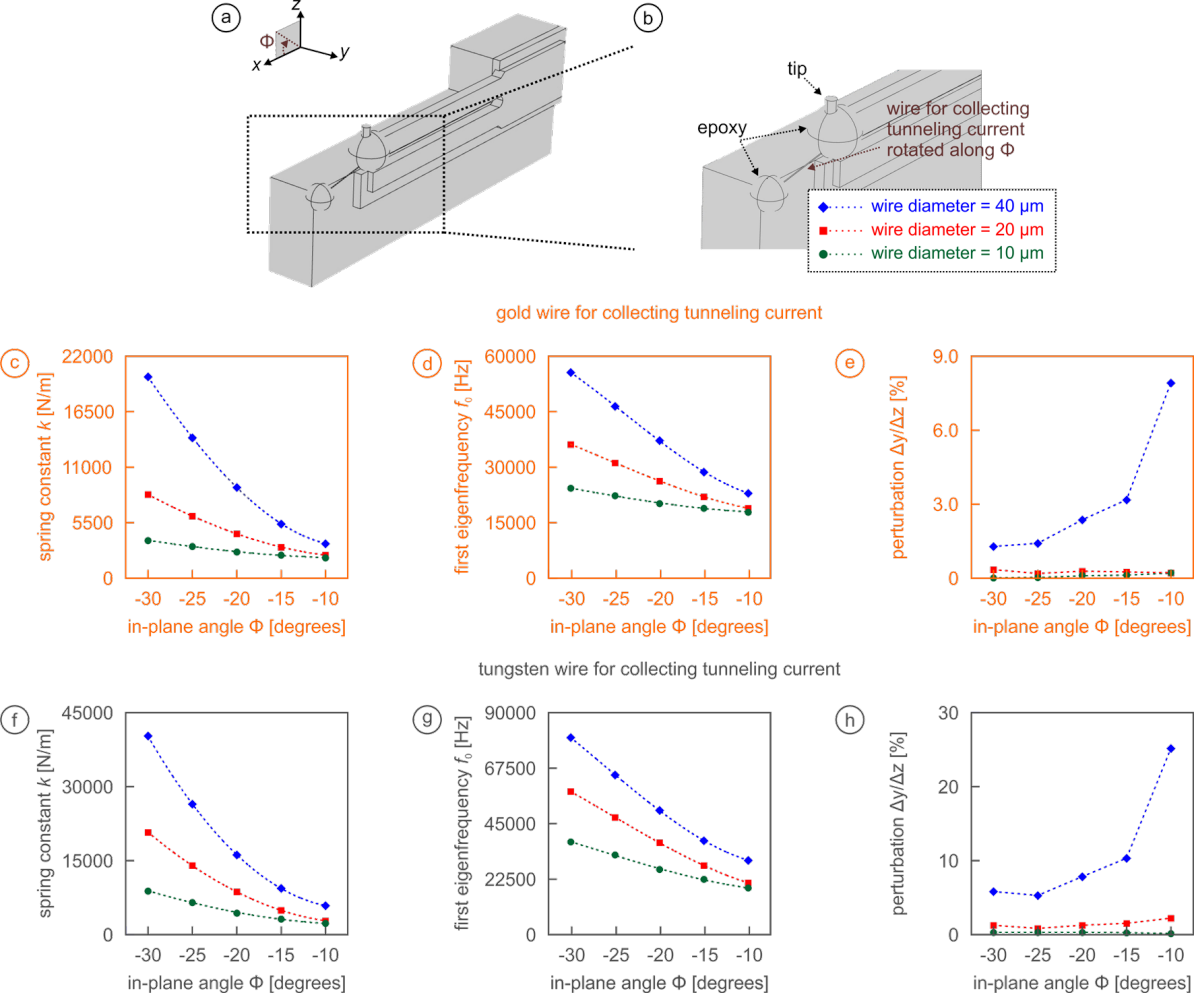
Effect of material choice (gold or tungsten), wire diameter (10 µm, 20 µm, or 40 µm), and in-plane angle 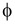 on spring constant *k*, eigenfrequency *f*_0_, and perturbation ∆*y*/∆*z* of the sensor. (a,b) Perspective view of the model geometry and definition of the in-plane angle 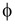. (c–h) Results from finite element modeling for gold (c–e) and tungsten (f–h) wires with three different diameters: 10 μm (green circles); 20 μm (red squares); and 40 μm (blue diamonds). For certain parameter sets, large increases from the values without wire are detected for *k* and *f*_0_, while the perturbation Δ*y*/Δ*z* remains acceptably small except for the thickest wire diameters.

For the first model configuration in Figure 5a,b, we assume that the wire, which can have three different diameters (10 µm, 20 µm, and 40 µm) and be made of two different metals (gold and tungsten), is attached to the Macor holder with a spherical blob of epoxy glue of 150 µm radius and runs to the tip that is symmetrically at the end of the prong (same configuration as in Figure 2a–c) with 225 µm radius and no tip tilt (φ = 0°). For this configuration, the location of the blob is moved downwards so that the angle between the horizontal line and the wire (the “in-plane angle” 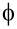) increases from 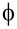 = −10° (smallest angle possible since the top of the Macor holder is lower than the prong) to 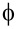 = −30° in increments of 5°. In this context, please note that (i) the length of the wire is always kept constant at 800 µm with the effect that the epoxy glue is moving slightly ‘inwards’ when moving downwards, and (ii) the size of the epoxy blob on the holder of 150 µm was chosen so that it is large enough that changing its size has only negligible influence on the obtained results.

Results for *k*, *f*_0_, and Δ*y*/Δ*z* are shown in Figure 5c–h. We first note that such an arrangement drastically increases the spring constant (Figure 5c,f), from the previous values of less than 2000 N/m [21,26,30–31,40–41] to values up to twenty times of that for the thickest tungsten wire. The angle 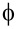 has a significant influence, increasing the spring constant up to the four-fold for 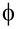 = −30° compared to 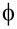 = −10°. The first eigenfrequency of the sensor assembly (Figure 5d,g) follows the same trends as the spring constant. Due to the stiffening of the spring, *f*_0_ may rise from below 20 kHz to over 80 kHz. In all cases, choosing thicker wire diameters or preferring the stiffer tungsten to the softer gold increases *k* and *f*_0_. Since *k* rises faster than *f*_0_ and Δ*f* 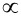 *f*_0_/*k*, any attempt should be made to keep both *k* and *f*_0_ as low as possible. An increase in the resonance frequency of the sensor can be also an indication of the effective stiffness of the assembly. Finally, the evolution of the perturbation Δ*y*/Δ*z* presents a most unusual behavior: While we find relatively low values (<1%) for both tungsten and gold wires that are 20 µm or smaller in diameter, wires featuring 40 µm diameter show dramatically higher values (up to 26% for tungsten), with the perturbation being the highest for the smallest in-plane angles 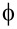 (Figure 5e,h). Together, the data presented in Figure 5 implies that one should employ the softest, thinnest wires possible if such connections for tunneling current should be added to avoid very notable effects on the sensing properties of the setup.

**Figure 6:**
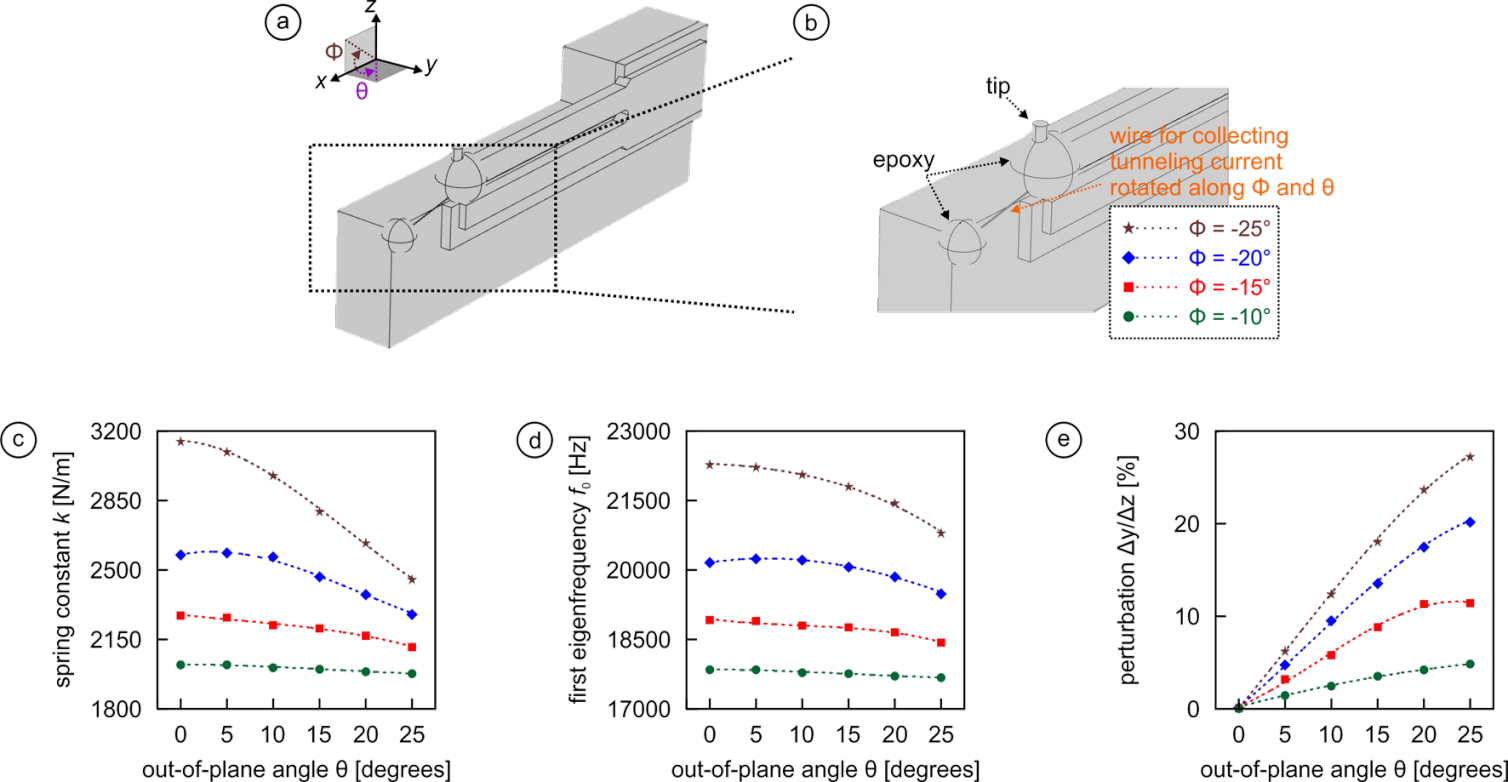
Effect of out-of-plane asymmetry (*x*–*y* plane) of the wire used to collect the tunneling current on *k*, *f*_0_, and ∆*y*/∆*z*. The use of a thin gold wire with 10 µm diameter is assumed, both the in-plane angle 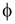 and the out-of-plane angle θ are defined in panels (a) and (b). Calculations for the out-of-plane angle θ run from 0 to 25°. For larger angles, the spherical 150 µm diameter epoxy blob securing the end of the wire to the holder would lose its contact with the holder. We show evolution of *k* (c), *f*_0_ (d), ∆*y*/∆*z* (e) for different in-plane angles 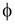 and different sets of the out-of-plane angle θ.

In Figure 6, we extend the configuration of Figure 5 by adding an out-of-plane component represented by the “out-of-plane angle” θ for four different values of 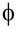 (−10°, green circles; −15°, red squares; −20°, blue diamonds; and −25°, brown stars). For simplicity, we focus on soft gold wires with the smallest diameter (10 µm) only. We find that larger values of θ cause the spring constant *k* to attenuate while for larger values of 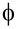, *k* increases (Figure 6c). The trends for the eigenfrequency are roughly the same (Figure 6d), but because the increases are again smaller as for the spring constant, the smallest values of both θ and 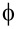 are generally most favorable. Most significantly, the perturbation ∆*y*/∆*z* increases gradually with the out-of-plane asymmetry and reaches 5% for 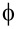 = −10° and 29% for 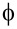 = −25° (Figure 6e). Since for a typical peak-to-peak oscillation amplitude of 2 Å a perturbation of 29% means an lateral motion of about 0.6 Å during the course of one oscillation cycle, this result reveals that any out-of-plane asymmetry of the wire used to collect the tunneling current should be kept minimal. As an alternative approach that avoids any wire-induced non-linearity, it has also become popular to use specialized three-electrode quartz beams that allow experimentalists to collect the tunneling current by means of a separate electrode [42]. In this case, however, cross-talk may be induced between different data channels due to the finite size of the electrodes.

## Conclusion

In this paper, we have provided a systematic study based on numerical calculations using finite element models of completely assembled qPlus sensors that include attached tips. Our numerical calculations reveal that a non-symmetric alignment of the tip attachment causes the spring constant to decrease and the perturbation of the ideal vertical oscillation behavior to increase while the eigenfrequency and quality factor experience only minor changes. Except for the case where the tip is mounted at half-length, changes are, however, small compared to configurations where the tip is contacted by an external wire for the collection of a tunneling current. Our analysis shows that even for the case where the thinnest, softest wire is being used (10 µm diameter gold wire), the spring constant may vary by a factor of two (between ca. 2000 N/m and ca. 4000 N/m in Figure 5c). This implies that the often-used assumption that the spring constant of a qPlus sensor is 2000 N/m may be an over-simplification for quantitative force spectroscopy [36], inducing a systematic error in such measurements. We also established that the more *k* and *f*_0_ depart from the values without wire connection, the smaller Δ*f* 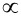 *f*_0_/*k* tends to be as *k* usually increases faster than *f*_0_ and that asymmetric wire connections may impose non-negligible lateral motions during an oscillation cycle, both of which negatively affect the sensing properties. Therefore, attention should be focused on using the softest, thinnest wires for establishing tip connections in qPlus sensors and to attach these soft wires in the most symmetric manner.
